# Cell-Based Methods for Determination of Efficacy for Candidate Therapeutics in the Clinical Management of Cancer

**DOI:** 10.3390/diseases6040085

**Published:** 2018-09-22

**Authors:** Jenna L. Gordon, Mark A. Brown, Melissa M. Reynolds

**Affiliations:** 1 Department of Chemistry, Colorado State University, Fort Collins, CO 80521, USA; jenna.short@colostate.edu; 2 Department of Clinical Sciences, Colorado State University, Fort Collins, CO 80521, USA; mark.brown@colostate.edu; 3 Department of Chemistry, Colorado State University, Campus Delivery 1872, Fort Collins, CO 80523, USA; 4 School of Biomedical Engineering, Colorado State University, Fort Collins, CO 80523, USA

**Keywords:** in vivo/in vitro correlation, bioassay approaches, pharmaceutical analysis

## Abstract

Determination of therapeutic efficacy is a major challenge in developing treatment options for cancer. Prior to in vivo studies, candidate therapeutics are evaluated using cell-based in vitro methods to assess their anti-cancer potential. This review describes the utility and limitations of evaluating therapeutic efficacy using human tumor-derived cell lines. Indicators for therapeutic efficacy using tumor-derived cell lines include cell viability, cell proliferation, colony formation, cytotoxicity, cytostasis, induction of apoptosis, and cell cycle arrest. Cell panel screens, 3D tumor spheroid models, drug-drug/drug-radiation combinatorial analysis, and invasion/migration assays reveal analogous in vitro information. In animal models, cellular assays can assess tumor micro-environment and therapeutic delivery. The utility of tumor-derived cell lines for efficacy determination is manifest in numerous commercially approved drugs that have been applied in clinical management of cancer. Studies reveal most tumor-derived cell lines preserve the genomic signature of the primary tumor source and cell line-based data is highly predictive of subsequent clinical studies. However, cell-based data often disregards natural system components, resulting in cell autonomous outcomes. While 3D cell culture platforms can counter such limitations, they require additional time and cost. Despite the limitations, cell-based methods remain essential in early stages of anti-cancer drug development.

## 1. Introduction 

Despite advances in technology and medicine, cancer remains one of the most lethal diseases in the world [1]. Lung cancer alone, the leading cause of worldwide cancer-related deaths, causes more than one million deaths per year [2,3]. Even after nearly a century of research on cancer treatments, more than one-third of all cancer patients in developed nations fail to survive five years post-diagnosis [4]. As the incidence of cancer continues to increase [5], attention must be placed on refining existing techniques and developing new methods to diagnose, prevent, and treat cancer patients. Although the best opportunity for achieving complete remission is early detection [6], many types of cancer do not manifest evident symptoms in the earliest stages [7]. As a result, it is important to develop and administer the most effective treatments possible for cancer patients at all stages.

**Figure 1:**
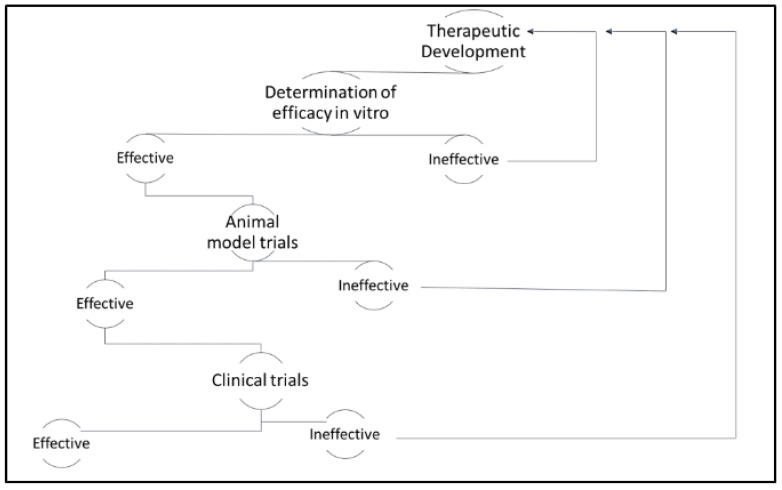
Diagram of therapeutic application post-therapeutic development. Therapeutics that are determined efficacious in vitro are applied in animal model trials. Therapeutics that are determined efficacious in animal model trials are applied in clinical trials. At any point, if the therapeutic is determined to be ineffective, researchers must return to the therapeutic development stage.

The development of therapeutics for the clinical management of cancer is traditionally defined in several distinct phases, including discovery, in vitro testing, pre-clinical animal studies, and clinical trials (see Figure 1). While the focus of the latter phases is to assess both safety and efficacy, most studies in the early phases of drug development focus on establishing efficacy alone. Tumor-derived cell lines have been the mainstay for anti-cancer drug discovery, and the assessment of in vitro, efficacy since the 1950s [8]. However, issues related to cross-contamination of cell lines and lack of translational relevance plagued early cell-based studies [9]. It was not until the establishment of the National Cancer Institute 60 (NCI-60) panel of human tumor-derived cell lines that cell-based efficacy studies became both economically feasible and translationally relevant. Shortly thereafter, the Japanese Foundation for Cancer Research established a similar panel of tumor-derived cell lines [9]. Both panels have been used extensively and have yielded thousands of candidate therapeutics. More recently, the Center for Molecular Therapeutics 1000 (CMT1000) platform of tumor-derived cell lines has been developed and validated to capture the greatest possible breadth of heterogeneity across cancer types [10]. This more comprehensive representation of human cancers has been more effective in predicting variation in clinical responses to treatment and it has ultimately paved the way for more efficient stratification of cancer patients according to the most suitable treatments. 

Once the sensitivity of certain tumor types has been established for a candidate anti-cancer therapeutic, researchers are able to scale back the breadth of cell types in order to focus their efficacy studies on the select cell lines, for which the drug exhibited the greatest potential. A range of indicators is commonly assessed at this point to gauge therapeutic efficacy including the impact of the drug on cell viability, cell proliferation, colony formation, cytotoxicity, cytostasis, induction of apoptosis, and cell cycle arrest [11,12,13,14,15,16,17,18]. To observe these impacts, a battery of in vitro assays may be employed, such as monoculture proliferation assays, 3D tumor spheroid models, drug-drug and drug-radiation combinatorial analysis, and invasion and migration assays. Cell-based studies can also serve as a platform for evaluating mechanism of action, impacts on invasion and migration, tumor hypoxia, cell-cell interactions, and cell-matrix interactions. 

In the determination of therapeutic efficacy, it is important to ascertain the appropriate method(s) to employ. This decision includes thorough evaluation of any pertinent analytical, clinical, and general considerations. Explicitly, important analytical considerations include detection sensitivity, data reproducibility, the ability to multiplex, reagent stability, and the number of cells present in a sample. Some of the clinical considerations are the cell line(s) of interest and the mode of action of the relevant anticancer pharmaceutical(s) (e.g., chemicals, antibodies, CAR-T cells). Additionally, some general considerations critical to assay selection include time, cost, ease of use, and instrument availability [19]. The section below provides an overview of common cell-based assays, which can be used to observe indicators of therapeutic efficacy. 

## 2. Cell-Based Assays for Determination of Therapeutic Efficacy

### 2.1. Cell Viability and Proliferation Assays

Cellular viability and proliferation assays are ubiquitously used to assess the effect of candidate anti-cancer therapeutics, including cytostatic and cytotoxic agents [12,13,14,15,16]. Cellular viability represents the number of healthy cells present in a population [20]. Cellular proliferation represents the ability of healthy cells to divide and create progeny [21]. Therefore, cell viability assays and cell proliferation assays are used to quantify the number of healthy cells in a population and/or the rate of growth of a population of cells [22]. This is accomplished by measuring markers of cell activity, such as metabolic activity, the number of cells present or divisions occurring within the population, ATP production, or DNA synthesis [21]. Often, these parameters are measured via colorimetric, binding, or staining assays. However, it can be difficult to distinguish between cytotoxicity and cytostasis using these methods alone, which can lead to ambiguous cell survival results [23]. Distinguishing these impacts is often accomplished using the adenine triphosphate-based tumor chemosensitivity assay (ATP-TCA) [24], or laser scanning cytometry [25]. Cytostatic agents are often employed as adjuvant therapeutics in tandem with a cytotoxic agent, such as a chemotherapeutic, to circumvent issues related to resistance and dose-limiting toxicity [13,16,26,27].

### 2.2. Colorimetric Assays

Metabolic activity is a common indicator of cell health. Thus, cell-based colorimetric assays are often employed in the determination of cellular metabolic activity. For example, tetrazolium salts and resazurin are reduced through mitochondrial dehydrogenase activity by viable cells [23]. As cells die or stop proliferating, there is a measurable change in the reduction of resazurin and tetrazolium salts [28,29,30]. The corresponding change in turnover rate can be analyzed via colorimetric and/or fluorescent detection [23]. Some of the most common tetrazolium salts used in cell-based metabolic studies are MTT (3-(4,5-dimethylthiazol-2-yl)-2,5-diphenyltetrazolium bromide), MTS (3-(4,5-dimethylthiazol-2-yl)-5-(3-carboxymethoxyphenyl)-2-(4-sulfophenyl)-2*H*-tetrazolium), XTT (2,3-bis-(2-methoxy-4-nitro-5-sulfophenyl)-2*H*-tetrazolium-5-carboxanilide), WST-1, and WST-8 (2-(2-methoxy-4-nitrophenyl)-3-(4-nitrophenyl)-5-(2,4-disulfophenyl)-2*H*-tetrazolium, monosodium salt) [23,31]. Due to the vast number of colorimetric assays, the advantages and disadvantages vary between individual analyses. Thus, the individual strengths and limitations of each assay are highlighted in Table 1. In general, colorimetric assays are easy to use, permit retrieval of high-throughput data, and provide a cost effective approach to determination of therapeutic efficacy. However, colorimetric assays often do not provide the ability to multiplex or obtain real-time measurements. Further, sufficient sensitivity may not be obtained when working with smaller samples of cells (<1000) [23].

Analysis of colorimetric assays generate absorbance values, which are an indirect measurement of cell viability. Treated samples are expressed as a percentage of 100% viable cells (absorbance of untreated sample—absorbance of cell media) [23]. Colorimetric assay results can produce a viability of zero, which does not necessarily indicate that every cell is dead, but instead indicates that there is no detectable change in absorbance between the cell media itself and the sample. 

### 2.3. Binding Assays

#### Neutral Red Uptake

The neutral red (3-amino-7-dimethylamino-2-methylphenazine hydrochloride) assay is another assay, commonly used to determine cell viability [34,35,36,37,38,39]. Similar to tetrazolium salts and resazurin, the neutral red (NR) assay is a colorimetric assay that allows quantitative determination of healthy cells through spectrophotometric detection [40]. Unlike the previously mentioned colorimetric assays, the NR assay depends on the ability of healthy cells to uptake NR within the lysosomes subsequent to exposure to a toxic substance [23]. NR is a weakly cationic dye that changes from an orange-red to deep red after forming bonds with anionic sites within the lysosomal matrix [41]. After exposure to a toxic substance, cell viability is expressed as a concentration dependent reduction of the NR uptake into the cell. This is possible because toxic substances alter the integrity of the cell, leading to a reduced uptake of NR. Although the NR assay is both rapid and sensitive, it does not definitively distinguish between cytotoxicity and cytostasis [23]. 

### 2.4. ATP Production

Another technique to analyze cellular viability in cell-based anti-cancer studies is the quantitation of ATP production [42,43,44,45]. The ATP production bioluminescence assay is based on the ability of luciferase to convert luciferin into oxyluciferin in an ATP-dependent reaction that generates light. Thus, the level of ATP correlates with the amount of light emission [23]. Cell viability can then be correlated to an increase in the overall amount of ATP produced in a population of exponentially growing cells [45,46]. This assay is particularly useful because it is sensitive enough to reproducibly detect the ATP production from a single mammalian cell [47]. An additional benefit is the large dynamic range of this assay, as concentration of ATP and cellular viability are directly proportional for cell numbers between one and one-hundred million cells [23]. However, as with other such assays, the ATP production assay does not allow for multiplexing and cannot distinguish between cytotoxicity and cytostasis. Additionally, accurate quantitation of ATP requires medium, long-term exposure to a toxic drug in vitro (48–72 h) [23].

### 2.5. Colony Formation Assays

Soft agar colony formation assays are often valuable in cancer research [13,15,16,48,49] because they allow for direct evaluation of tumor progression in vitro in a cellular environment that mimics in vivo conditions [48]. Colony formation assays operate on the principal that healthy cells require contact with the extracellular matrix present in the body to grow and divide. Conversely, transformed or malignant cells are able to grow and divide without regard to the surrounding environment [17]. As a result, transformed cells form colonies within the semi-solid agar matrix. When counted as colony forming units (CFUs), the number of colonies formed provides a quantitative assessment of the malignant potential of individual cell lines. Subsequent to exposure of a colony-forming cell line to an anti-tumor therapeutic on agar, a decrease in colony-formation with respect to increasing therapeutic concentration is indicative of therapeutic efficacy [50]. 

### 2.6. Cytotoxicity Assays

#### 2.6.1. Staining and Imaging

Cytotoxicity assays are used to assess both live and dead cells after treatment with a therapeutic [23]. Staining techniques are often used in cytotoxicity assays to quickly visualize the presence of live and dead cells through fluorescence microscopy [14,51,52,53]. Propidium iodide is a fluorescent, membrane impermeable stain that binds to DNA of dead cells by intercalating between bases with little or no sequence preference [54]. Since propidium iodide has a molecular weight of only 668.4 Da, it can bind to dead cells with minimal disruption to the cell membrane [51]. Live cells with intact cell membranes will exclude the dye and exhibit little to no fluorescence [55]. The application of a live cell counter stain (membrane permeable DNA stain) facilitates the assessment of both live and dead cells simultaneously [56]. Advantages of propidium iodide staining include the ability to multiplex cell samples, rapid analysis, and ease of use. Conversely, this method does not reveal the mechanism of cell death [23] and data reproducibility relies on the control of variations in exposure time and camera/software settings [56]. 

#### 2.6.2. LDH Cytotoxicity Assay

Another commonly used cytotoxicity assay is the lactate dehydrogenase (LDH) release assay, which allows for the rapid and sensitive short-term (1 h post cell death) detection of cytotoxicity in various cell types [23]. The LDH assay resembles colorimetric cell viability assays in light of the mechanistic and detection similarities. When a cell loses membrane integrity, it releases LDH, which is then used as a catalyst to promote a two-step reaction. The first step is the oxidation-reduction reaction between NAD+ and lactate. This is followed by the reduction of a tetrazolium salt (INT) to a colored formazan. The colored formazan product can be detected colorimetrically through the absorbance maximum at 490–520 nm. In order to accurately determine cytotoxicity using this assay, it is important to account for the inherent LDH activity that occurs within the cell culture medium. Additionally, if the candidate therapeutic induces cell death intracellularly and without the loss of plasma membrane integrity, the LDH assay will not detect the occurrence of cell death [23]. One potential benefit of the LDH cytotoxicity assay is the potential to distinguish between cell death and growth inhibition with a modified LDH-based cytotoxicity assay [57].

### 2.7. Cell Apoptosis Assays

Apoptosis is a reliable indicator of cancer therapeutic efficacy and can be evaluated based on a variety of biochemical and morphological indicators [58]. The most common methods for observing apoptosis in cancer cells are described below. 

#### 2.7.1. DNA Fragmentation

DNA fragmentation assays have been a mainstay for observing apoptosis [36,57,58,59,60,61]. As cells undergo apoptosis, nuclear DNA fragmentation occurs, providing a detectable parameter for the observation of late stage apoptosis. Traditionally, DNA fragmentation has been observed via laddering on agarose gels through assays, such as conventional agarose gel electrophoresis (CAGE) and pulsed-field gel electrophoresis (PFGE). Specifically, CAGE allows for the detection of low-molecular-weight fragments and can be refined to include radioactive end labeling, permitting the detection of small quantities of fragmented DNA. Alternatively, PFGE allows for the detection of high-molecular-weight DNA fragments [62]. However, results obtained through these assays can be ambiguous, because homogenate preparation and necrotic cells can also produce DNA fragments. Although these methods are simple to perform, large cell samples (at least 1 million cells) are necessary to achieve reliable results [23]. 

In attempt to increase DNA fragmentation assay utility, the Single Cell Gel Electrophoresis (Comet) assay has been developed [24]. In 1984, the first microelectrophoretic method for visualization of DNA damages in individual mammalian cells was introduced by Ostling and Johansen [63]. Six years later, the Ostling and Johansen method was refined to enhance single-cell sensitivity and simultaneously coined the Comet assay [64]. Advantageously, the Comet assay requires relatively few cells (~1000 cells) and allows for distinction of heterogeneity within a sample population [23,65]. Nevertheless, relatively small sample populations can be detrimental when large variation exists within that population. Further, the Comet assay does not provide any information about the size of the DNA fragments produced [65]. Additionally, the assay can be adapted from its original basic conditions to allow for measurement of cells at lower pH (termed “neutral” assay) [23,65].

Subsequent to the development of the Comet assay, terminal deoxynucleotidyl transferase dUTP nick end labeling (TUNEL) was developed for the visualization of nuclei containing DNA fragments [66,67]. Specifically, TUNEL allows for detection of DNA fragments in situ through labeling of the free 3′-hydroxyl termini on DNA fragments. However, in its early-nineties form, the TUNEL assay was considered nonspecific, prohibiting consistent distinction between DNA fragments produced by apoptotic or necrotic cells [54]. Since then, the sensitivity and selectivity of the TUNEL assay have been optimized to further ensure accurate distinction between apoptosis and necrosis [68]. Advantageously, TUNEL can also be combined with Annexin V to comprise a more robust assay that is capable of distinguishing apoptosis and necrosis. Since Annexin V binding is reported to occur prior to DNA fragmentation, it is capable of detecting necrotic or early apoptotic cells that exhibit a negative response from TUNEL [69]. 

#### 2.7.2. Caspase Activation

Since caspase activation is a hallmark of cell apoptosis [23], numerous assays have been developed to detect activation of apoptosis-related caspases. One of the most common of these employs the Western blot to measure caspase activation [11,12,13,14,15,16,27,70,71]. However, Western blotting is only semi-quantitative, does not allow for multiplexing [72], and does not demonstrate the type of cell undergoing apoptosis [23]. Alternatively, numerous commercial kits exist to monitor apoptosis-related caspases, including the Caspase-Glo 3/7 Assay [73], caspase 3 colorimetric assay kit [52], and CellEvent caspase-3/7 Green Detection Reagent [74]. The use of assay kits can be advantageous when a limited number of analyses are planned. Additionally, assay kits allow for analysis of individual caspases signaling various apoptotic processes. However, more extensive studies economically compel in-house preparation of assay components [75].

#### 2.7.3. Flow Cytometry

Flow cytometry presents a reliable multi-parameter detection technique for observation of cell apoptosis [76]. In flow cytometry, it is possible to measure the size and complexity of cells, as well as fluorescence. For example, a treated sample of cells can be stained with a live and/or dead cell stain and fluorescing antibodies against markers of apoptosis. Often, stains, such as PI, 7-aminoactinomycin (7AAD), 4′,6-diamidino-2-phenylindole (DAPI), or Annexin V, are applied singularly or in combination [62]. Additionally, fluorescent dyes (fluorochromes) are commonly linked to mono- or polyclonal antibodies. The application of antibodies in flow cytometry is beneficial for the detection of different apoptotic pathways [77]. For example, Anti-caspase 3 allows for the detection of caspase 3 dependent apoptosis. Similarly, numerous antibodies exist for the detection of different apoptotic pathways, including other members of the caspase family, PARP, and BRdU [78].

After an incubation period, the sample is introduced into the flow cytometer in conjunction with a sheath fluid that is flowing at a different rate than the sample suspension. The varying flow rates between the two fluids allows for hydrodynamic focusing of the sample suspension, which directs the sample cells to pass through the laser light source in a single file line [77]. As the cells pass through the laser light source, they exhibit forward and side scattered light, and potentially fluorescence. Forward scattered light corresponds to the size of the cell with more forward scatter corresponding to a larger cell. Similarly, side scattered light corresponds to the complexity of the cell, with more side scatter corresponding to a more complex cell. Finally, fluorescence at a specific wavelength is observed from live or dead cells, depending on the type of cell stain and/or antibodies used. Flow cytometry provides researchers with the specific mechanism of cell death, highly reproducible data, due to the possibility of single cell analysis, and the ability to rapidly multiplex cell samples [79]. 

### 2.8. Cell Cycle Arrest Assays

Cell cycle arrest assays operate on the foundation of cellular regulation through cell cycle checkpoints. Cancer therapeutics can be designed to target specific cell cycle checkpoints in neoplastic cells in order to induce cell death. The phase at which a therapeutic causes neoplastic cell arrest in vitro can indicate its potential efficacy in vivo. Although there are various methods through, which cell cycle checkpoints can be analyzed, such as [3*H*]-thymidine incorporation, BrdU incorporation, and microscopy, cell cycle arrest assays are often analyzed using flow cytometry [80]. 

Flow cytometry permits single-cell quantification of stained DNA to indicate the percentage of cells existing in each cell cycle phase. Through fluorescence detection, flow cytometric data is provided in the form of a histogram, indicating the percentage of cells in the G_0_ and G_1_ phase (2N DNA content), the S phase (between 2N and 4N DNA content), and the G2/M phase (4N DNA content) [81]. Apoptotic cells can further be distinguished between the cells in various phases, because the DNA content (and fluorescence intensity) are less than that in the G0/G1 phase. Multiparameter analysis of cell cycle phase is also possible using flow cytometry, through the analysis of RNA content and DNA susceptibility to denaturation under various environmental parameters [80].

### 2.9. 3D Cell Culture Systems

The methods identified above were originally developed using monolayer cultures. Such systems lack the rich heterogeneity of tumor micro-environments and the corresponding analyses are limited to cell autonomous outcomes that fail to account for impacts on tumor-stromal interaction, angiogenesis and other such factors of a. natural system With greater emphasis on the importance of tumor three-dimensionality (3D) and their corresponding microenvironments with regard to therapeutic efficacy, the advanced stages of the in vitro testing phase often includes 3D cell culture systems to more closely model physiological conditions [82,83,84]. 3D cultures have the added utility of observing characteristics, such as variations in polarity, invasive potential, and matrix independent survival. However, substratum rigidity is an additional concern as it has been shown to be involved in regulation of cell processes [85].

#### 2.9.1. Multicellular Tumor Spheroids

Among 3D culture systems, the multicellular tumor spheroids (MTCS) system is one of the best characterized [86,87]. The MTCS system facilitates high fidelity simulation of tumor micro-environments and in vivo growth conditions with regard to pathophysiology and observed responsiveness to therapeutics [86,87,88]. It has been widely employed to evaluate a range of impacts associated with candidate therapeutics, including cell-cell interactions, cell-matrix interactions, chemical gradients, metabolic gradients, and resistance [89]. 

#### 2.9.2. Hollow Fibre Assays

The hollow fiber assay is a technological innovation built upon prior techniques for microencapsulation and subsequent cultivation of cells [90,91,92]. The hollow fiber assay involves a 1–2 day, in vitro culture incorporating a panel of tumor cell lines contained in biocompatible hollow fibers and the subsequent subcutaneous implantation of the fibers in mice. Mice are treated with the candidate therapeutic for a period of several days and the fibers are subsequently removed to facilitate cell viability assays. Thus, this method can assess therapeutic efficacy along with the ability of a drug to reach its target in vivo, and it can provide preliminary data related to the safety of the therapeutic. The hollow fiber assay is further advantageous for its savings on time and required quantity of therapeutic, relative to traditional in vivo assays. It also allows for the in vivo examination of therapeutic efficacy related to tumor-derived cell lines that would not otherwise grow in an animal model. Given the strong correlation between efficacy in hollow fiber assays and efficacy observed with human xenografts, the hollow fiber system also serves as an effective way to screen potential therapeutics prior to costly xenograft experiments [90]. 

### 2.10. Cell-Based Systems for Evaluating Combinatorial Efficacy

Development of combinatorial treatments has been historically slow and often involved trial-and-error in clinical settings. However, advancements in cell-based systems has, more recently, facilitated the use of cell-based screening platforms to evaluate combinatorial efficacy in high-throughput systems. For example, one such experiment evaluated 600 commercially-approved therapeutics in a combinatorial analysis of almost 100,000 groupings for combinatorial efficacy in human, lung tumor-derived cells [93]. Studies of this kind have the potential to uncover synergism among therapeutic applications that may prevent the development of resistance common to single-drug approaches. 

## 3. Conclusions

Tumor-derived cell lines largely preserve the genomic signature of the primary tumors, from which they were sourced [94,95] and data obtained using such cell lines is highly predictive of subsequent clinical outcomes [10,96]. Therefore, in attempt to improve the efficacy of anti-cancer drugs entering clinical trials, it is essential to determine the efficacy of each drug in vitro as accurately as possible. This process begins with proper assay understanding, selection, and execution. Cell viability assays are useful for indirect determination of the number of live cells present after treatment with a therapeutic. Colony formation assays directly illuminate the ability of a therapeutic to inhibit tumor proliferation. Cytotoxicity assays present a direct method for the visualization of live and dead cells after treatment with a therapeutic. Cell apoptosis assays allow for direct quantification of the number of apoptotic cells after treatment with a therapeutic. Lastly, cell cycle arrest assays allow for indirect quantification of the number of apoptotic cells that deceased at specific phases within the cell cycle. Although each assay presented encompasses various strengths and limitations, no single method proves therapeutic efficacy. It is important to strategize and employ multiple methods in order to ensure accurate and reliable results. 

The importance of cell-based studies for evaluating therapeutic efficacy is underscored by the growing number of commercially approved drugs and biologics for the clinical management of cancer. However, there are major limitations to analyses of monocultured cell lines. Such cell lines often lack the rich heterogeneity of tumor micro-environments and the corresponding analyses are limited to cell autonomous outcomes that fail to account for impacts on tumor-stromal interaction, angiogenesis and other such factors of a natural system. While 3D cell culture platforms have been employed to respond to such limitations of monocultures, they do so at the expense of greater time and cost. Despite these limitations, cell-based methods remain essential in the early stages of anti-cancer drug development.

## Figures and Tables

**Table 1:** Comparison of the advantages and disadvantages of determining cell viability and/or proliferation using various colorimetric assays. Advantages are indicated via check marks (√). Disadvantages are indicated through an x (χ). Multiple check marks (√) indicate the degree of advantage of one assay in comparison to others [22,23,30,31,32,33].

Assay	Real-Time Measurements	Sensitivity	Multiplexing	Water-Soluble	Additional Intermediates Not Required	Ease-of-Use	Cost
MTT	χ	√	χ	χ	√	√	√
MTS	χ	√	χ	√	χ	√√	√
XTT	χ	√	χ	√	χ	√√	√
WST-1	χ	√	χ	√	χ	√√	√
WST-8	√	√√	√	√	√ (Already incorporated)	√√√	√
Resazurin	√	√√	√	√	√	√√	√√
